# Efficient production of phenyllactic acid in *Escherichia coli* via metabolic engineering and fermentation optimization strategies

**DOI:** 10.3389/fmicb.2024.1457628

**Published:** 2024-08-23

**Authors:** Weibin Wu, Maosen Chen, Chenxi Li, Jie Zhong, Rusheng Xie, Zhibin Pan, Junhan Lin, Feng Qi

**Affiliations:** ^1^Fujian Vocational College of Bioengineering, Fuzhou, China; ^2^Engineering Research Center of Industrial Microbiology of Ministry of Education, College of Life Sciences, Fujian Normal University, Fuzhou, China

**Keywords:** metabolic engineering, *Escherichia coli*, phenyllactic acid, synthetic biology, dissolved oxygen feedback fed-batch

## Abstract

Phenyllactic acid (PhLA), an important natural organic acid, can be used as a biopreservative, monomer of the novel polymeric material poly (phenyllactic acid), and raw material for various medicines. Herein, we achieved a high-level production of PhLA in *Escherichia coli* through the application of metabolic engineering and fermentation optimization strategies. First, the PhLA biosynthetic pathway was established in *E. coli* CGSC4510, and the phenylalanine biosynthetic pathway was disrupted to improve the carbon flux toward PhLA biosynthesis. Then, we increased the copy number of the key genes involved in the synthesis of the PhLA precursor phenylpyruvic acid. Concurrently, we disrupted the tryptophan biosynthetic pathway and enhanced the availability of phosphoenolpyruvate and erythrose 4-phosphate, thereby constructing the genetically engineered strain MG-P10. This strain was capable of producing 1.42 ± 0.02 g/L PhLA through shake flask fermentation. Furthermore, after optimizing the dissolved oxygen feedback feeding process and other conditions, the PhLA yield reached 52.89 ± 0.25 g/L in a 6 L fermenter. This study successfully utilized metabolic engineering and fermentation optimization strategies to lay a foundation for efficient PhLA production in *E. coli* as an industrial application.

## 1 Introduction

Phenyllactic acid (PhLA) is a small-molecule natural organic acid found in pickles and natural honey (Armaforte et al., 2006). It can be polymerized into poly (phenyllactic acid), a novel material with good thermal stability, strong ductility, excellent ultraviolet absorption, and chemical resistance, making it a research focus in fields such as biology, chemistry, and energy (Nguyen et al., 2011; Erickson et al., 2012). Moreover, PhLA is a novel and effective antibacterial and antifungal preservative, providing a viable solution to microbial contamination and reducing food safety-related hazards (Schnürer and Magnusson, 2005; Valerio et al., 2016). It is a crucial raw material for producing drugs such as englitazone, nonprotein amino acids, hypoglycemic agents, and anti-human immunodeficiency virus agents (Urban and Moore, 1992; Weckwerth et al., 2000). Additionally, PhLA inhibits platelet aggregation in blood vessels, dilating them to increase coronary flow and combat myocardial hypoxia (Wu et al., 2007). These characteristics of PhLA provide promising application prospects in the pharmaceutical industry.

Currently, there are two main approaches of PhLA synthesis: chemical and microbial syntheses. Chemical synthesis of PhLA generates many by-products, such as phenylpyruvic acid (PhPA), and also involves complex technical routes, harsh reaction conditions, serious environmental pollution, and difficulties in subsequent separation (Bustos et al., 2018). Microbial synthesis of PhLA offers several advantages, such as mild reaction conditions, easy availability of fermentation substrates, lesser environmental pollution, relatively low cost, and good development prospects (Kawaguchi et al., 2015). Therefore, microbial production has become an attractive alternative method. In lactic acid bacteria, PhLA is a by-product of phenylalanine metabolism. Phenylalanine undergoes transamination to produce PhPA under the catalysis of deaminase, with α-ketoglutaric acid as an amino acceptor. Then, PhPA is converted to PhLA by a dehydrogenase (Mu et al., 2009; Prasuna et al., 2012).

**Figure 1:**
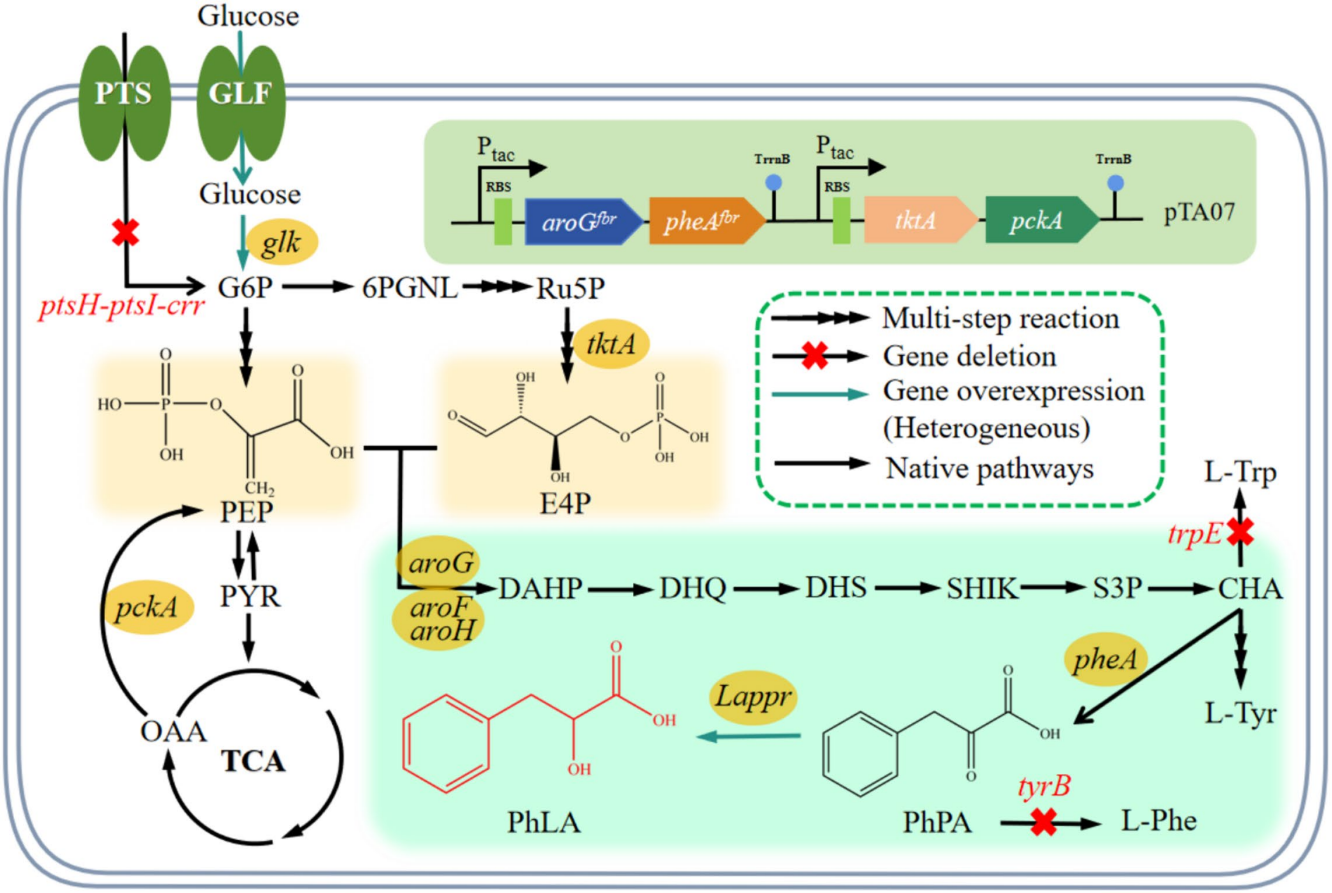
PhLA biosynthetic pathway and relevant metabolic reactions in *E. coli*. The conversion reaction of PhPA into PhLA was constructed by introducing the *Lactobacillus ppr* gene encoding phenylpyruvate reductase in *E. coli*. Abbreviations for metabolites: G6P, glucose-6-phosphate; 6PGNL, glucose 6-phosphate endolipid; Ru5P, ribulose-5-phosphate; E4P, erythrose-4-phosphate; PEP, phosphoenolpyruvate; PYR, pyruvate; TCA, tricarboxylic acid; OAA, oxaloacetic acid; DAHP, 3-deoxy-D-arabinoheptulosonic acid 7-phosphate; DHQ, 3-dehydroquinate; DHS, 3-dehydroshikimate; SHIK, shikimate; S3P, SHK-3-phosphate; CHA, chorismate; L-Trp, L-tyrptophan; L-Tyr, L-tyrosine; L-Phe, L-phenylalanine; PhPA, phenylpyruvate; PhLA; phenyllactic acid. Genes and enzymes: *glf*, glucose facilitator; *glk*, glucokinase; *tktA*, transketolase; *pckA*, PEP carboxykinase; aroF, *aroG*, and *aroH*, DAHP synthase AroF, AroG, and AroH, respectively; *pheA*, chorismate mutase/prephenate dehydratase; *trpE*, anthranilate synthase; *tyrB*, tyrosine aminotransferase; *Lappr*, phenylpyruvate reductase. PTS, glucose phosphotransferase system encoded by the *ptsG*, *ptsH*, *ptsI*, and *crr* genes.

With the development of metabolic engineering and synthetic biology, biocatalysis technology has been widely applied, and constructing microbial cell factories for the heterologous synthesis of PhLA has attracted increasing attention (Liu et al., 2015). Phenylalanine, PhPA, and α-ketoglutaric acid are precursors for synthesizing PhLA. By exogenously adding these precursors, microbial conversion can be used to produce PhLA and thus increase its yield (Weber and Zannoni, 1966; Valerio et al., 2016). Phenylalanine is converted into PhLA through transamination and reduction, with the former reaction being the probable rate-limiting step in PhLA biosynthesis. In a previous study, α-ketoglutaric acid was added during the culture of *Lactobacillus plantarum* TMW1.468, improving the transamination of phenylalanine and markedly enhancing the PhLA yield (Vermeulen et al., 2006). According to another study, lactate dehydrogenase and formate dehydrogenase were co-expressed to engineer *Escherichia coli* for PhLA production; this strain could convert 82.8 mmol/L PhPA to 79.6 mmol/L PhLA (Zheng et al., 2015). Some researchers utilized *E. coli* to co-express L-lactate dehydrogenase (L-LDH) derived from *L. plantarum* and glucose dehydrogenase (GDH) derived from *Bacillus megaterium*, using PhPA as the substrate for whole-cell catalytic synthesis of L-PhLA, ultimately achieving a final product concentration of 103.8 mmol/L, approximately 17.2 g/L (Zhu et al., 2017). Similarly, PhPA was used as a substrate for bioconversion, achieving a PhLA yield of 20.5 g/L (Luo et al., 2020). However, there are limited recent reports of the *de novo* synthesis of PhLA, with most reports focusing on whole-cell catalytic synthesis using PhPA as a substrate (Zheng et al., 2015; Zhu et al., 2017). The high cost of PhPA makes biocatalysis more expensive. In a previous study, phenylpyruvate reductase (encoded by *pprA*) from *Wickerhamia fluorescens* TK1 was expressed in a high-phenylalanine—producing *E. coli* strain, and the engineered strain produced 29 g/L PhLA under optimized fermentation conditions using glucose as the substrate (Fujita et al., 2013). The heterologous expression of phenylpyruvate reductase in *E. coli* to construct a PhLA biosynthetic pathway was considered feasible (Figure 1). However, in addition to the enhanced expression of phenylpyruvate reductase, the supply levels of initial substrates—phosphoenolpyruvate (PEP) and erythrose-4-phosphate (E4P)—are critical for the efficient synthesis of PhLA in *E. coli* (Figure 1). Exploring efficient *de novo* synthesis of PhLA from low-cost raw materials is an urgent issue for future research.

A PhLA biosynthetic pathway was established in the phenylalanine-producing *E. coli* CGSC4510 for the efficient *de novo* synthesis of PhLA. The genes *aroG^fbr^* and *pheA^fbr^*, crucial for the PhLA synthesis pathway, were overexpressed in engineered *E. coli*, and the tryptophan synthesis pathway was weakened to increase metabolic flux and enhance PhLA yield. Additionally, the central metabolic pathway and glucose transport system were modified to improve the supply of the precursors PEP and E4P. Finally, the glucose-feeding process was optimized based on the dissolved oxygen (DO)-feedback, and the potential of the engineered *E. coli* to produce PhLA in a 6 L fermenter was evaluated. This study provides a successful example of applying *E. coli* for the efficient synthesis of PhLA.

## 2 Materials and methods

### 2.1 Media and culture conditions

The media used in this study include Luria-Bertani (LB) medium (10 g/L peptone, 5 g/L yeast extract, 10 g/L NaCl), seed medium (10 g/L NaCl, 15 g/L peptone, 10 g/L yeast extract, 2 g/L glucose), and fermentation medium (20 g/L glucose, 4 g/L yeast extract, 3 g/L MgSO_4_·7H_2_O, 5.5 g/L (NH_4_)_2_SO_4_, 3 g/L K_2_HPO_4_, 0.7 g/L tyrosine, 0.7 g/L phenylalanine, 0.35 g/L tryptophan, 0.5 g/L sodium citrate, 0.6 g/L sodium glutamate, 0.05 g/L FeSO_4_, 0.004 g/L MnSO_4_, 0.5 g/L fumaric acid, 0.001 g/L VB_1_, 0.001 g/L VB_3_, 0.0003 g/L VB_8_).

For shake flask fermentation, a single colony was inoculated into 5 mL of LB medium containing appropriate antibiotics and cultivated overnight at 37°C and 220 rpm. The culture was then inoculated into 20 mL of seed medium at a 5% (v/v) ratio and cultivated overnight at 37°C and 220 rpm. The seed culture was then inoculated into fermentation medium at a 10% (v/v) ratio and cultivated at 37°C and 200 rpm for 48 h. Ampicillin (100 mg/L) was added during cultivation to promote plasmid retention. When the culture reached an OD_600_ of 0.6, IPTG was added to a final concentration of 0.1 mM to induce recombinant protein expression. Each strain was cultured in triplicate.

### 2.2 Chemicals and reagents

All chemicals and reagents were of analytical grade and HPLC grade, purchased from Sigma-Aldrich Inc. (Shanghai, China) or Beijing Chemical Works (Beijing, China). Q5 High-Fidelity DNA Polymerase, Gibson Assembly Cloning Kit, and restriction enzymes were purchased from New England BioLabs (Beijing, China). Plasmid extraction and purification kits were purchased from Shandong Sparkjade Biotechnology Co., Ltd. (Shandong, China). Primers for PCR amplification were synthesized by Biosune (Fuzhou, China).

### 2.3 Construction of plasmids and strains

All genes used in this study were either amplified from genomic DNA or synthesized by General Biosystems (Anhui, China) with codon optimization. Genes were amplified using primer pairs tac-aroG^fbr^-F/R, tac-pheA^fbr^-F/R, and tac-GA-F/R, and then ligated with linearized plasmid pTrc99a to construct plasmids pTA03, pTA04, and pTA05 to enhance the carbon flux of PhLA. To further optimize expression, the *tktA* gene was amplified with the primer pair tac-tktA-F/R and ligated with the linearized plasmid pTA05 using the Gibson assembly method, resulting in plasmid pTA06. Similarly, plasmid pTA07 was constructed to overexpress *pckA*. The primers used for plasmid construction in this study are listed in Supplementary Table S1, and all constructed plasmids are shown in Table 1.

**Table 1:** Plasmids and strains used in this study.

Plasmids	Description	Source or reference
pTrc99a	Ptrc promoter, AmpR, *E. coli* expression vector	This lab
pTarget	pMB1 StrR sgRNAs with or without donor DNAs	This lab
pCas	Pcas-cas9 ParaB-Red lacIq Ptrc-sgRNA-pMB1 kanR	This lab
pTA03	pTrc99a, P_tac_-*aroG^fbr^*	This study
pTA04	pTrc99a, P_tac_-*pheA^fbr^*	This study
pTA05	pTrc99a, P_tac_-*aroG^fbr^*-*pheA^fbr^*	This study
pTA06	pTrc99a, P_tac_-*aroG^fbr^*-*pheA^fbr^*, P_tac_-*tktA*	This study
pTA07	pTrc99a, P_tac_-*aroG^fbr^*-*pheA^fbr^*, P_tac_-*tktA*-*pckA*	This study
pTargetΔ*tyrB*::*Lappr*	pTarget, sgRNA, N20, *tyrB*-LH, *Lappr*, *tyrB*-RH	This study
pTargetΔ*tyrB*::*EcyiaE*	pTarget, sgRNA, N20, *tyrB*-LH, *EcyiaE*, *tyrB*-RH	This study
pTargetΔ*tyrB*::*WfpprA*	pTarget, sgRNA, N20, *tyrB*-LH, *WfpprA*, *tyrB*-RH	This study
pTargetΔ*trpE*	pTarget, sgRNA, N20, *trpE*-LH, *trpE*-RH	This study
pTargetΔ*ptsH-ptsI-crr*::*gif*-*gik*	pTarget, sgRNA, N20, *ptsH-ptsI-crr*-LH, *gif*-*gik*, *ptsH-ptsI-crr*-RH	This study

Strain	Description	Source or reference
*E. coli* CGSC4510	Derived from *E. coli* K-12, Hfr (PO1), λ−, el4-, tyrA4, relA1, spoT1, thiE1	This lab
MG-P1	*E. coli* CGSC4510Δ*tyrB*::*Lappr*	This study
MG-P2	*E. coli* CGSC4510Δ*tyrB*::*EcyiaE*	This study
MG-P3	*E. coli* CGSC4510Δ*tyrB*::*WfpprA*	This study
MG-P4	*E. coli* CGSC4510Δ*tyrB*::*Lappr* containing the plasmid pTA03	This study
MG-P5	*E. coli* CGSC4510Δ*tyrB*::*Lappr* containing the plasmid pTA04	This study
MG-P6	*E. coli* CGSC4510Δ*tyrB*::*Lappr* containing the plasmid pTA05	This study
MG-P7	*E. coli* CGSC4510Δ*trpE*Δ*tyrB*::*Lappr* containing the plasmid pTA05	This study
MG-P8	*E. coli* CGSC4510Δ*trpE*Δ*tyrB*::*Lappr* containing the plasmid pTA06	This study
MG-P9	*E. coli* CGSC4510Δ*trpE*Δ*tyrB*::*Lappr* containing the plasmid pTA07	This study
MG-P10	MG-P9Δ*ptsH-ptsI-crr*::*gif*-*gik*	This study

The CRISPR/Cas9 technique was performed mainly according to previously reported methods (Shen et al., 2022). Phenylpyruvate reductase (encoded by *ppr*) from *Lactobacillus* sp. CGMCC 9967, glycolaldehyde reductase (encoded by *yiaE*) from *E. coli*, and phenylpyruvate reductase (encoded by *pprA*) from *W. fluorescens* TK1 were integrated into the amino transferase (encoded by *tyrB*) site of the *E. coli* CGSC4510 genome and expressed under the inducible promoter tac, resulting in recombinant strains MG-P1, MG-P2, and MG-P3. Additionally, anthranilate synthase (encoded by *trpE*) was knocked out to weaken the competing pathway. Furthermore, to increase the availability of PEP, a precursor for phenylalanine lactate biosynthesis, glucose permease (encoded by *glf*) and glucose kinase (encoded by *glk*) from *Zymomonas mobilis* were integrated in tandem into the critical gene loci (*ptsH-ptsI-crr*) of the carbohydrate phosphotransferase system (PTS) in the *E. coli* genome and expressed under the inducible promoter tac. Accordingly, all constructed strains in this study are named and listed in Table 1.

### 2.4 Dissolved oxygen feedback fed-batch control strategy

Feedback feeding fermentation strategy is often used to achieve high-concentration production of the target product. During the feedback fed-batch fermentation process, when glucose is used as the sole carbon source, dissolved oxygen (DO) directly reflects glucose utilization (Park et al., 2011; Lv et al., 2020). When glucose is consumed, the oxygen uptake rate accompanied by a decrease in cell growth rate will lead to an increase in DO value. When glucose is supplemented, the increase in oxygen uptake rate that accompanies the increased cell growth rate will lead to a decrease in the DO value. It is commonly believed that the advantage of controlling the glucose supplementation rate through DO-feedback fed-batch control lies in its ability to prevent both glucose excess and DO limitation. By employing this method, an increase in cell growth and product yield can be achieved (Jing et al., 2018).

The dissolved oxygen feedback fed-batch control strategy in this study: 3 L fermentation medium was fermented in a 6 L bioreactor (Bioflo310, New Brunswick Scientific) for 48 h. The medium used was the same as the aforementioned fermentation medium, and 300 mL seed culture was added to the 3 L fermentation medium. The initial stirring speed was 200 rpm, with an aeration rate of 0.5 vvm, and the pH of the culture was maintained at 7.0 by automatically adding 25% ammonia water. The cultivation temperature was 37°C, and the dissolved oxygen was maintained at 5–10%, 10–20%, 20–30%, and 30–40% during fermentation. Sampling from the bioreactor is done by placing a centrifuge tube at the fermenter sampling port and using the fermenter tank pressure to discharge the fermentation broth out of the sampling port. Glucose was used as the carbon source in the fermentation medium, and the glucose concentration was controlled at 0.1–1.0% by feeding 60% glucose solution, with an initial glucose concentration of 2%. When the DO concentration exceeded the set value, the fermentation control system would feed 60% glucose solution into the fermentation medium. When the DO concentration dropped to the set saturation value, the automatic feeding system would stop, indicating the restoration of cell viability due to nutrient supplementation.

### 2.5 Analytical methods

The yield of PhLA was detected and analyzed using high-performance liquid chromatography (HPLC, Waters Alliance e2695, United States). An Inert Sustain AQ-C18 column (5 μm, 4.6 × 250 mm) was used, with a column temperature of 30°C, an injection volume of 20 μL, and a flow rate of 1.0 mL/min. The detection wavelength was 210 nm. The mobile phase A is 0.05% trifluoroacetic acid aqueous solution, and mobile phase B is 0.05% trifluoroacetic acid methanol solution. The gradient was 40% B to 80% B from 0 to 15 min, maintained at 80% B from 15 to 16 min, and decreased from 80% B to 40% B from 16 to 18 min. Glucose concentration in the culture was measured using an enzyme electrode glucose sensor (BF-4, Oji Scientific Instruments, Hyogo, Japan). OD_600_ was measured using a UV–visible spectrophotometer (UV-1800, Shimadzu, Japan) to monitor cell growth, and dry cell weight (DCW) was calculated based on a pre-calibrated relationship (OD = 0.33 g/L DCW) (Wu et al., 2018).

## 3 Results

### 3.1 Construction of the PhLA biosynthetic pathway in *Escherichia coli*

**Figure 2:**
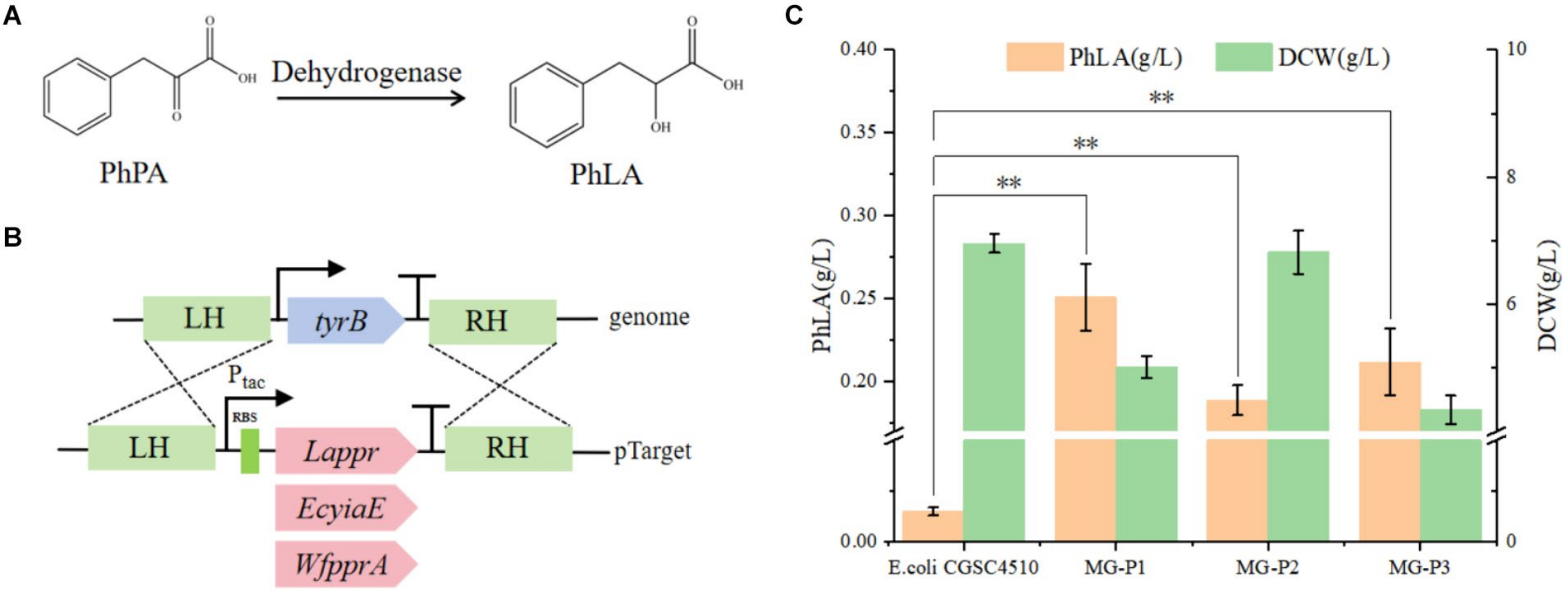
Construction of the PhLA biosynthesis pathway in *E. coli*. **(A)** The reduction of phenylpyruvate to PhLA catalyzed by dehydrogenase. **(B)** Schematic representation of homologous recombination of dehydrogenases from different sources into the *TyrB* locus. **(C)** PhLA production titers in recombinant *E. coli* strains MG-P1, MG-P2, and MG-P3, expressing *Lappr*, *EcyiaE*, and *WfpprA*, respectively. ^**^p < 0.001. The data shown are the averages of three independent experiments with the standard deviations.

PhPA can be converted to PhLA catalyzed by phenylpyruvate reductase (Xu et al., 2016). In this study, we constructed a PhLA biosynthetic pathway in *E. coli* by overexpressing phenylpyruvate reductase (encoded by *ppr*) from *Lactobacillus* sp. CGMCC 9967, glycolaldehyde reductase (encoded by *yiaE*) from *E. coli*, and phenylpyruvate reductase (encoded by *pprA*) from *W*. *fluorescens* TK1 (Figure 2A). From our laboratory collection, we utilized *E. coli* CGSC4510, which is capable of producing phenylalanine as the starting strain. In the phenylalanine biosynthetic pathway of *E. coli*, PhPA is converted to phenylalanine by aminotransferase (TyrB), whose expression is regulated by TyrR (Liu et al., 2018). Therefore, to enable *E. coli* to biosynthesize PhLA, we employed the CRISPR/Cas9 technology to integrate the genes *Lappr*, *EcyiaE*, and *WfpprA* under the control of the tac promoter into the TyrB locus of the *E. coli* CGSC4510 chromosome, resulting in the strains MG-P1, MG-P2, and MG-P3, respectively (Figure 2B). This transformation disrupted the phenylalanine biosynthetic pathway while constructing the PhLA biosynthetic pathway. In shake flask fermentation, MG-P1 expressing *Lappr* had the highest PhLA yield, reaching 0.251 ± 0.02 g/L, which was 24.7 and 15.5% more than that of MG-P2 (0.189 ± 0.01 g/L) and MG-P3 (0.212 ± 0.02 g/L), respectively (Figure 2C). Additionally, the residual quantity of phenylalanine is presented in Supplementary Figure S1. These results indicate that *La*PPR had enhanced activity than the other two enzymes, showing greater catalytic activity toward PhPA, thus converting more PhPA to PhLA. Consequently, we successfully constructed a PhLA biosynthetic pathway in *E. coli*, resulting in the MG-P1 strain, which has a robust capability to produce PhLA and was used for further experiments.

### 3.2 Improving the metabolic flux of the PhLA synthesis pathway

PhPA serves as the primary precursor of PhLA, with its intracellular concentration directly affecting the production of PhLA. In *E. coli*, aromatic amino acids (such as phenylalanine) are naturally produced via the shikimate pathway (Figure 1). The first reaction in this pathway is the condensation of PEP and E4P, catalyzed by 3-deoxy-D-arabino-heptulosonate-7-phosphate (DAHP) synthases. This reaction is the first rate-limiting step during the synthesis of aromatic amino acids. The three isoenzymes of DAHP synthases—AroG, AroH, and AroF—are subject to feedback inhibition by their respective end products—phenylalanine, tryptophan, and tyrosine. The activity of DAHP synthases determines the carbon flux into the shikimate pathway (Bongaerts et al., 2001). AroF and AroG contribute to 20 and 80% of the total enzyme activity, respectively (Tribe et al., 1976). The second rate-limiting step occurs during the synthesis of PhPA, catalyzed by the bifunctional enzyme chorismate mutase—prephenate dehydratase (PheA), which plays a crucial role in the production of PhPA and is subject to feedback inhibition by phenylalanine (Liu et al., 2018). Therefore, optimizing these rate-limiting steps is essential for improving the production of PhLA. For efficient PhLA production, it is necessary to overexpress *aroG^fbr^* (Kikuchi et al., 1997) and *pheA^fbr^* (Zhou et al., 2010) in *E. coli*.

**Figure 3:**
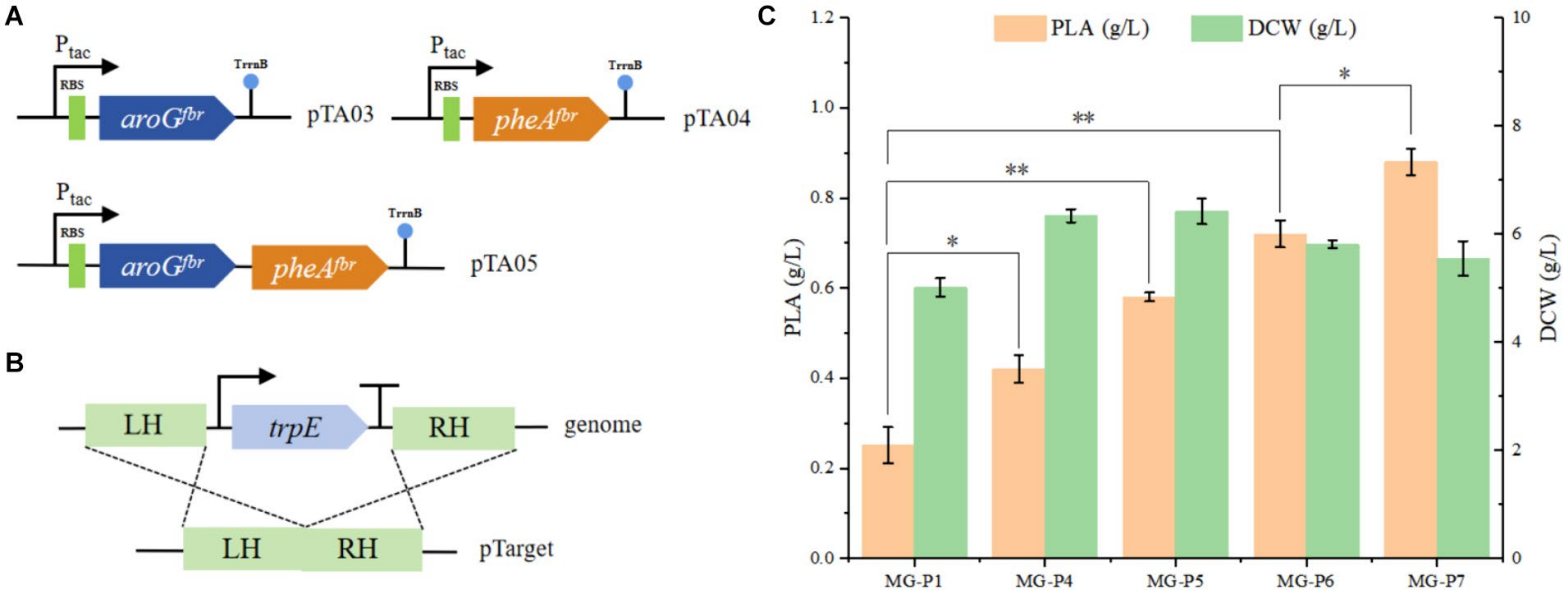
To enhance the production of PhLA, the rate-limiting enzymes of the PhLA synthesis pathway were overexpressed, and the tryptophan synthesis pathway was depleted. **(A)** Plasmids pTA03, pTA04, and pTA05 were constructed to overexpress the *aroG^fbr^* and *pheA^fbr^* individually and simultaneously, respectively. **(B)** Diagram of the *trpE* knockout. **(C)** Titers of PhLA produced by the recombinant *E. coli* strains MG-P1, MG-P4, MG-P5, MG-P6, and MG-P7. ^*^p < 0.05 and ^**^p < 0.001. The data shown are the averages of three independent experiments with the standard deviations.

We constructed the plasmids pTA03, pTA04, and pTA05 (Figure 3A) to overexpress *aroG^fbr^* and *pheA^fbr^* individually or simultaneously. These plasmids were transformed into the MG-P1 strain, resulting in the recombinant strains MG-P4, MG-P5, and MG-P6. In shake flask fermentation, compared with MG-P1 strain, PhLA production by MG-P4, MG-P5, and MG-P6 increased by 67, 131, and 186%, respectively. Overexpressing *pheA^fbr^* alone was more effective than overexpressing *aroG^fbr^* alone, and their combined overexpression had the most pronounced effect on the PhLA yield, with its titer reaching 0.72 ± 0.03 g/L (Figure 3C). The overexpression of *aroG^fbr^* and *pheA^fbr^* increased the metabolic flux into the phenylalanine biosynthesis pathway, thereby enhancing the production of PhLA. Additionally, in the aromatic amino acid metabolic pathway, chorismic acid can diverge into two metabolic branches: one leading to phenylalanine and tyrosine and the other to tryptophan. Inhibition of the tryptophan biosynthetic pathway can direct more carbon flux toward the phenylalanine metabolic branch (Bang et al., 2016). Therefore, to further enhance PhLA production, we knocked out *trpE* from the MG-P6 strain, resulting in the MG-P7 strain (Figure 3B). In shake flask fermentation, PhLA production was enhanced by 22%, achieving a titer of 0.88 ± 0.03 g/L in MG-P7 strain (Figure 3C). This result demonstrates that inhibiting the tryptophan biosynthetic pathway prevents carbon flux loss, thereby further enhancing PhLA production.

### 3.3 Enhancing the supply of PEP and E4P and their impact on PhLA production

The availability of PEP and E4P as initial substrates is a crucial factor for achieving efficient production of PhLA. In *E. coli*, PEP is a key intermediate in glucose transport and a precursor for the biosynthesis of pyruvate and oxaloacetate (Figure 1). The systems and enzymes involved in the production and consumption of PEP mainly include the PTS system, PEP synthase, PEP carboxykinase, PEP carboxylase, and pyruvate carboxylase (Bongaerts et al., 2001). The PTS system consumes approximately 50% of the PEP, leaving only a small amount for the synthesis of aromatic amino acids (Gosset, 2005). Replacing the PTS system with the glucose transport system that does not rely on PEP can improve the efficiency of aromatic amino acid synthesis by reducing PEP consumption and thus increasing the carbon flux into the aromatic amino acid biosynthetic pathway (Liu et al., 2017; Chen et al., 2018). Glucose permease (Glf) from *Pseudomonas putida* has a excellent glucose uptake efficiency (Parker et al., 1995). The synthesis of one molecule of phenylalanine requires the consumption of two molecules of PEP and one molecule of E4P. E4P can be produced under the catalysis of transketolase (encoded by *tktA*) or transaldolase (encoded by *talB*), with TktA being more effective than TalB in directing carbon flux toward the synthesis of aromatic amino acids (Lu and Liao, 1997).

**Figure 4:**
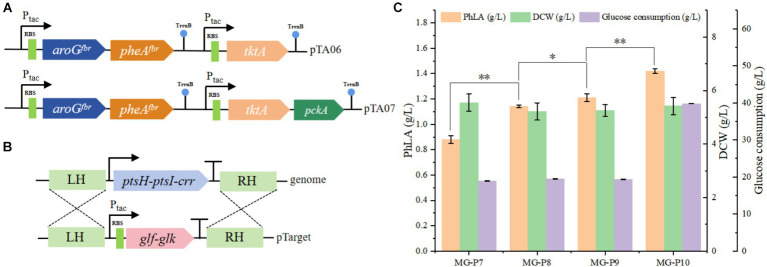
Enhancing the utilization efficiency of the precursor substances PEP and E4P in *E. coli* to improve PhLA production. **(A)** Construction of plasmids pTA06 and pTA07 for overexpressing the *tktA* and *pckA* genes. **(B)** Diagram of the homologous recombination of Glk and Glf from *Z. mobilis* into the ptsH-ptsI-crr locus. **(C)** PhLA production by recombinant *E. coli* strains MG-P8, MG-P9, and MG-P10, with MG-P10 cultured in fermentation medium containing 40 g/L glucose. ^*^p < 0.05 and ^**^p < 0.001. The data shown are the averages of three independent experiments with the standard deviations.

The impact of enhanced precursor supply on PhLA production was investigated by further overexpressing *tktA* in the pTA05 plasmid, resulting in the pTA06 plasmid (Figure 4A). This plasmid was employed to transform the MG-P7 strain, producing the MG-P8 strain. Shake flask fermentation showed that elevating E4P supply during MG-P8 culture effectively enhanced PhLA production, resulting in a titer of 1.14 ± 0.01 g/L, a 30% enhancement, compared to the control strain (Figure 4C). Similarly, to increase PEP supply, we overexpressed phosphoenolpyruvate carboxykinase (encoded by the *pckA*) in the MG-P8 strain (Figure 4A), generating the MG-P9 strain, which produced 1.21 ± 0.03 g/L PhLA, indicating an increase of 6.1%. Additionally, we knocked out *ptsH-ptsI-crr* in MG-P9 to disrupt the native PTS system in *E. coli*. We then complemented these loci with the genes encoding Glf and glucose kinase (Glk) from *Z. mobilis* to restore the growth capability, creating the MG-P10 strain (Figure 4B). After culturing this strain in a fermentation medium with 40 g/L glucose for 48 h, the PhLA yield reached 1.42 ± 0.02 g/L, with a glucose consumption of 39.8 g/L. Compared to the control strain, the PhLA titer and the conversion rate of glucose to PhLA were remarkably improved (Figure 4C; Supplementary Figure S2). This finding indicates that the co-expression of *glf* and *glk* enhanced the glucose uptake efficiency, thereby improving the growth and PhLA production abilities of the strain. These results suggest that the combined action of TktA, PckA, Glk, and Glf elevated the intracellular availability of PEP and E4P, significantly boosting PhLA production.

### 3.4 Enhanced PhLA production through DO-feedback feeding control strategy

**Figure 5:**
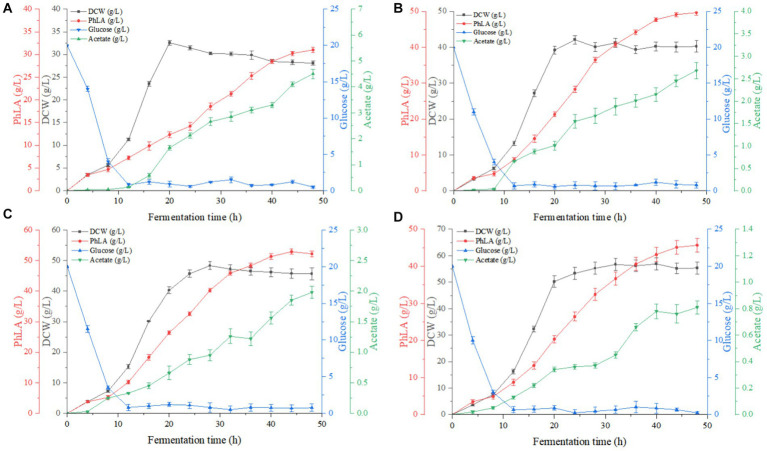
Fed-batch fermentation of recombinant *E. coli* for PhLA production based on dissolved oxygen (DO)-feedback control strategy in the 6 L bioreactor. **(A)** DO-feedback setting value = 5–10%. **(B)** DO-feedback setting value = 10–20%. **(C)** DO-feedback setting value = 20–30%. **(D)** DO-feedback setting value 30–40%. The data shown are the averages of three independent experiments with the standard deviations.

Based on the results of the shake flask fermentation of the engineered strain, MG-P10 was selected for fed-batch fermentation in a 6 L fermenter to evaluate its productivity and optimize the fermentation process. Previous reports have described the advantages of DO-feedback feeding in process control, which can suppress substrate feedback inhibition and DO supply limitations, thereby increasing the yield of the target product (Jing et al., 2018). Although providing excess glucose under oxygen-limited conditions during fermentation could improve PhLA yield (Kawaguchi et al., 2019), detailed reports on the effects of different levels of DO-feedback feeding on PhLA production in *E. coli* are still lacking. Therefore, we further explored the DO-feedback feeding strategy in a 6 L bioreactor to enhance the PhLA yield. Figure 5 and Table 2 show the changes in glucose concentration, DCW, PhLA titer, and acetic acid accumulation under various DO control levels. At a DO range of 20–30%, the PhLA titer, DCW, and glucose-to-PhLA yield reached 52.89 ± 0.25 g/L, 48.32 g/L, and 0.225 g/g (Figure 5C). Its PhLA titer is 6% higher than when the DO value is set in the range of 10–20% (Figure 5B). As the DO setpoint increased, there was a marked decline by up to 82% in the formation of the by-product acetic acid after 48 h of cultivation (Figures 5A,D). In *E. coli*, metabolic overflow occurs with excess glucose in the medium due to increased glucose consumption, leading to more by-products like acetic acid. This observation might be due to two reasons: first, the 20–30% DO-feedback feeding strategy avoided excessive glucose intake and insufficient DO supply; second, some acetic acid might have been metabolized by the strain to activate the TCA cycle, meeting its growth needs (Park et al., 2011; Kawaguchi et al., 2019). Compared to the other by-products, acetic acid severely inhibits *E. coli* growth and target product synthesis, thus reducing the fermentation performance (Kawaguchi et al., 2019). Under the enhanced DO-feedback feeding strategy of 30–40%, the DCW, PhLA titer, and glucose-to-PhLA yield of the engineered strain were 56.89 g/L, 45.68 ± 0.21 g/L, and 0.177 g/g, respectively (Figure 5D). Under these conditions, although the acetic acid content was only 0.81 g/L, the glucose conversion rate was 0.177, 21.3% lower than at the 20–30% DO setting, despite a 17.7% increase in DCW. These observations suggest that a part of the glucose was used for cell growth, and the relatively high DO level might have partially oxidized the raw materials and reduced productivity (Zhu et al., 2015). Additionally, this phenomenon could be due to the inhibition of glycolysis by the 20–30% DO level, restricting the energy required for PhLA biosynthesis. Under oxygen-limited conditions, the expression of *tktA*, *aroF*, *aroG*, and *aroH* in the shikimate pathway is upregulated, providing more E4P as a starting material for this pathway (Kawaguchi et al., 2019). Therefore, in this study, an appropriate DO-feedback feeding strategy could also address the problem of maintaining DO levels as cell density increases in the later stages of fermentation. These results indicate that maintaining DO-feedback feeding at 20–30% is more suitable for fermentation-based PhLA production, and optimizing the DO level is crucial for high PhLA yield.

**Table 2:** Comparison of dissolved oxygen DO-feedback feeding strategy on fed-batch fermentation of PhLA by *E. coli*.

	Dissolved oxygen (DO) level			
	5–10%	10–20%	20–30%	30–40%
Glucose consumption (g)	685.3 ± 0.15	969.1 ± 0.21	936.5 ± 0.26	1035.2 ± 0.29
Maximum titer of PhLA (g/L)	31.01 ± 0.22	49.68 ± 0.26	52.89 ± 0.25	45.68 ± 0.21
Maximum DCW (g/L)	32.56 ± 0.16	42.11 ± 0.12	48.32 ± 0.23	56.89 ± 0.26
Maximum acetate titer (g/L)	4.5 ± 0.13	2.68 ± 0.10	1.98 ± 0.12	0.81 ± 0.13
Average PhLA yield (g/L/h)	0.646 ± 0.13	1.035 ± 0.25	1.102 ± 0.16	0.951 ± 0.15
Glucose conversion (g/g)	0.181 ± 0.08	0.205 ± 0.11	0.225 ± 0.13	0.177 ± 0.06

## 4 Discussion

Herein, we constructed an efficient microbial cell factory using *E. coli* for the synthesis of PhLA. We also performed a series of rational and systematic optimizations of the PhLA biosynthetic pathway to achieve sustainable and green production. We disrupted the phenylalanine synthesis pathway in *E. coli* CGSC4510; heterologously expressed *Lappr*, *EcyiaE*, and *WfpprA*; and Reduction of PhPA to PhLA, thereby establishing the PhLA biosynthetic pathway in *E. coli*. Among these genes, *Lappr* encoded proteins exhibited the higher catalytic activity, enabling the engineered strain MG-P1 to produce 0.251 ± 0.02 g/L PhLA after 48 h of shake flask culture. Studies evaluating the effects of *pprA*, *gxrA*, *ycdW*, and *yiaE* on PhLA production indicated that *pprA* expression can enhance PhLA production more efficiently (Fujita et al., 2013). Here, we discovered a phenylpyruvate reductase, *La*PPR, with higher enzymatic activity, which has higher catalytic efficiency than *Wf*PPRA, possibly because the catalytic efficiency of *La*PPR is greater than that of *Wf*PPRA when using PhPA as substrate, which enables it to convert more PhPA into PhLA. The PhLA synthesis pathway is complex and tightly regulated, with DAHP synthases and PheA being the key rate-limiting enzymes. By overexpressing *aroG^fbr^* and *pheA^fbr^* and knocking out *trpE*, which is a crucial enzyme of the tryptophan synthesis pathway, we increased the metabolic flux. Consequently, the engineered *E. coli* strain MG-P7 achieved a PhLA titer of 0.88 ± 0.03 g/L via shake flask fermentation, indicating a 2.5-fold increase compared with that achieved using the MG-P1 strain. These results confirm that *aroG^fbr^* and *pheA^fbr^* are critical factors in determining the biosynthesis of PhLA, and their co-expression can significantly improve the PhLA yield, consistent with the finding of a previous study (Zhang et al., 2013). The increased PhLA yield is likely due to the overexpression of *aroG^fbr^*, which enhances the carbon flux from the central metabolism into the shikimate pathway, as well as the overexpression of *pheA^fbr^*, which channels more chorismic acid into the PhLA pathway. The disruption of *trpE* further prevents the loss of metabolic flux, ultimately leading to the efficient accumulation of PhLA (Kikuchi et al., 1997; Zhou et al., 2010; Bang et al., 2016). The supply of the precursors PEP and E4P as well as the intensity of the condensation reaction directly determine the metabolic flux of the shikimate pathway and yield of PhLA. We explored the impact of enhancing the supply of E4P and PEP on PhLA production by simultaneously overexpressing *tktA* and *pckA* and constructing a glucose transport system independent of PEP to replace the PTS system. Co-overexpression of *glf*, *glk*, *pckA*, and *tktA* in *E. coli* with a disrupted PTS system markedly enhanced the intracellular supply of PEP and E4P, resulting in a PhLA yield of up to 1.42 ± 0.02 g/L. This strategy not only improved the strain’s ability to produce PhLA but also enhanced glucose utilization. According to a previous study, the overexpression of *tktA* in *E. coli* slightly improved DAHP accumulation but not the DAHP conversion rate (Tyagi et al., 2017). Moreover, overexpression of *tktA* could disrupt central metabolism and glucose uptake (Ikeda and Katsumata, 1999). Overexpression of PEP synthase alone had no remarkable impact on tryptophan synthesis (Patnaik et al., 1995). These findings suggest that focusing metabolic engineering solely on increasing the accumulation of either of the substances in PEP and E4P may exacerbate the imbalance between the two, leading to the waste of carbon sources and ultimately failing to significantly improve synthesis efficiency. Therefore, synergistically elevating the supply levels of essential precursors is crucial for promoting PhLA accumulation. Finally, through the optimization of the DO-feedback feeding process, the engineered strain MG-P10 achieved a maximum PhLA titer of 52.89 ± 0.25 g/L in a 6 L fermenter when DO was maintained at a level of 20–30%, glucose-to-PhLA yield reached 0.225 g/g. This result indicates that optimal DO levels are essential for enhancing PhLA synthesis. In summary, this study successfully utilized an engineered *E. coli* strain for the *de novo* synthesis of PhLA, providing a foundation for its industrial production.

## 5 Conclusion

This study produced PhLA in *E. coli* via metabolic engineering and a DO-feedback feeding control strategy. By introducing phenylpyruvate reductase into *E. coli*, we constructed the biosynthetic pathway for PhLA. This pathway was enhanced by overexpressing the vital rate-limiting enzymes, weakening the tryptophan biosynthetic pathway, and increasing the supply of key precursors. Finally, PhLA accumulation in the engineered *E. coli* was further improved through optimization of the DO-feedback feeding control strategy. These results indicate that *E. coli* is an efficient chassis microorganism for the production of PhLA. This study demonstrates the potential of metabolic engineering and fermentation optimization strategies for efficient PhLA synthesis.

## Data Availability

The datasets presented in this study can be found in online repositories. The names of the repository/repositories and accession number(s) can be found in the article/Supplementary material.
