# Supplementary material for: Efficient production of phenyllactic acid in Escherichia coli via metabolic engineering and fermentation optimization strategies
Source: Front Microbiol. 2024 Aug 23;15:1457628. doi: 10.3389/fmicb.2024.1457628 (PMC11377314; doi:10.3389/fmicb.2024.1457628)
Supplement: Supplementary file 1 [file Data_Sheet_1.docx]

**Figure S1**. The production of L-Phe titers by recombinant *E. coli* MG-P1, MG-P2 and MG-P3. **P < 0.001. The data shown are the averages of three independent experiments with the standard deviations.

**Figure S2**. The production of PhLA by recombinant *E. coli* MG-P10 in fermentation media containing 20 g/L and 40 g/L glucose, respectively. *P < 0.05. The data shown are the averages of three independent experiments with the standard deviations.

**Supplementary Table 1. Primers Used in This Study**

| **Primers** | **Sequences (5'-3')** |
| --- | --- |
| tac-ppr-F | AATTAATCATCGGCTCGTATAATGAAGGAGAtgatgaagattctaaacagct |
| tac-ppr-R | ttatcagaccgcttctgcgtttcagtatccgcgcgtgagat |
| tac-yiaE-F | AATTAATCATCGGCTCGTATAATGAAGGAGATGAAGCCGTCCGTTATCCTC |
| tac-yiaE-R | tatcagaccgcttctgcgttTTAGTCCGCGACGTGCGGATTC |
| tac-pprA-F | ATTAATCATCGGCTCGTATAATGAAGGAGATGAAAAAGCCTCAGGTCCTT |
| tac-pprA-R | tatcagaccgcttctgcgttTCAAACTACAAGATTCATTTC |
| pTf-V-F | CACATATGATATATTCATATtttttttgaattctctagag |
| pTf-V-R | AGACGCCATAAAGAATGATGgcaccgactcggtgccactt |
| TyrB-L-F | aagtggcaccgagtcggtgcCATCATTCTTTATGGCGTCTCGCC |
| TyrB-L-R | CGAGCCGATGATTAATTGTCAAGCGATGGTTCTCCAGGTTTACG |
| TyrB-R-F | ctcagtcgaaagactgggccttTGCAGGAAAGCAGGCTGGAAT |
| TyrB-R-R | gactctagagaattcaaaaaaaATATGAATATATCATATGTGAATAGCAACAAAACGAC |
| TyrB-N20-F | GGCGGAAGCGCGCCTGAATGgttttagagctagaaatagcaag |
| TyrB-N20-R | CATTCAGGCGCGCTTCCGCCactagtattatacctaggactga |
| tac-aroG^fbr^-F | ggaaacagaccatggaattcTTGACAATTAATCATCGGCTCGTATAATGAAGGAGATGAATTATC |
| tac-aroG^fbr^-R | tagaggatccccgggtaccgagctcTTACCCGCGACGCGCTTTTACTG |
| pTA03-F | GCAGTAAAAGCGCGTCGCGGGTAAgagctcggtacccggggatcc |
| pTA03-R | ACGAGCCGATGATTAATTGTCAAgaattccatggtctgtttcctgtgtg |
| tac-pheA^fbr^-F | TTGACAATTAATCATCGGCTCGTATAATGAAGGAGATGACATCGGAAAACCCGTT |
| tac-pheA^fbr^-R | gaggatccccgggtaccgagctcTCAGGTTGGATCAACAGGCACTAC |
| pTA04-F | GTGCCTGTTGATCCAACCTGAgagctcggtacccgggga |
| pTA04-R | CATTATACGAGCCGATGATTAATTGTCAAgaattccatggtctgtttcctgtgt |
| tac-GA-F | caggaaacagaccatggaattcTTGACAATTAATCATCGGCTCGTATAATGA |
| tac-GA-R | tcgactctagaggatccccgTCAGGTTGGATCAACAGGCACTA |
| pTA05-F | TAGTGCCTGTTGATCCAACCTGAcggggatcctctagagtcgacc |
| pTA05-R | ACGAGCCGATGATTAATTGTCAAgaattccatggtctgtttcctgtgtg |
| trpE-L-F | aagtggcaccgagtcggtgcGACCGCGCGTCACTGCGCCG |
| trpE-L-R | ATCGAGCAGCAGAATGTCAGCCATGTTATTCTCTAATTTTGTTCAAAAAAAAGCCC |
| trpE-R-F | TGAACAAAATTAGAGAATAACATGGCTGACATTCTGCTGCTCG |
| trpE-R-R | tagagaattcaaaaaaaTGTTCCGGCTTCAGCTCG |
| pTf-trpE-F | TGGCGAGCTGAAGCCGGAACAtttttttgaattctctagagtcgacctgc |
| pTf-trpE-R | AGGGCGGCGCAGTGACGCGCGGTCgcaccgactcggtgccac |
| trpE-N20-F | ACTGGATAACGCCCTGCCTGgttttagagctagaaatagcaagttaaaataagg |
| trpE-N20-F | CAGGCAGGGCGTTATCCAGTactagtattatacctaggactgagctagct |
| trpE-Y-F | CAAGGGCCGCCAGCTGGGTA |
| trpE-Y-R | CGGCGTTCATATCAAGATTAATACCGAA |
| tac-tktA-F | gggatccTTGACAATTAATCATCGGCTCGTATAATGAAGGAGATGTCCTCACGTAAAGAG |
| tac-tktA-R | ttgcatgcctgcaggtcgactctagaTTACAGCAGTTCTTTTGCTTTCGC |
| pTA06-F | AAGCAAAAGAACTGCTGTAAtctagagtcgacctgcaggcatg |
| pTA06-R | TCCTTCATTATACGAGCCGATGATTAATTGTCAAggatccccgTCAGGTTGGAT |
| pckA-F | AAAGCAAAAGAACTGCTGTAAAAGGAGATGCGCGTTAACAATGGTTTGAC |
| pckA-R | gcatgcctgcaggtcgactctagaTTACAGTTTCGGACCAGCCG |
| pTA07-F | GCGGCTGGTCCGAAACTGTAAtctagagtcgacctgcaggca |
| pTA07-R | ACCATTGTTAACGCGCATCTCCTTTTACAGCAGTTCTTTTGCTTTCGC |
| ptsH-ptsI-crr-L-F | agtggcaccgagtcggtgcAGATTAAACCTGGCCCGCATA |
| ptsH-ptsI-crr-L-R | AGCCGATGATTAATTGTCAATGTATTTCCCCAACTTATAGGTTTAGTGTT |
| ptsH-ptsI-crr-R-F | ggctcagtcgaaagactgggccttTTCTTGCCGCAGTGAAAAATGG |
| ptsH-ptsI-crr-R-R | cgactctagagaattcaaaaaaaTGGTGCGATTCCGGACGAATGG |
| pTf-ptsH-ptsI-crr-F | ACCATTCGTCCGGAATCGCACCAtttttttgaattctctagagtcgacctgc |
| pTf-ptsH-ptsI-crr-R | TTATGCGGGCCAGGTTTAATCTgcaccgactcggtgcc |
| ptsH-ptsI-crr-N20-F | GGTCGGCAGAAATTTTTTTCgttttagagctagaaatagcaagttaaaataaggct |
| ptsH-ptsI-crr-N20-R | GAAAAAAATTTCTGCCGACCactagtattatacctaggactgagctagctg |
| ptsH-ptsI-crr-Y-F | GCATGAAAGGCGCAATCCAAA |
| ptsH-ptsI-crr-Y-R | GACTGCCAGAATCAGGTAAAGTTTCG |
